# Production and Characterization of Recombinant Wild Type Uricase from Indonesian Coelacanth (*L. menadoensis*) and Improvement of Its Thermostability by In Silico Rational Design and Disulphide Bridges Engineering

**DOI:** 10.3390/ijms20061269

**Published:** 2019-03-13

**Authors:** Sakda Yainoy, Thanawat Phuadraksa, Sineewanlaya Wichit, Maprang Sompoppokakul, Napat Songtawee, Virapong Prachayasittikul, Chartchalerm Isarankura-Na-Ayudhya

**Affiliations:** 1 Department of Clinical Microbiology and Applied Technology, Faculty of Medical Technology, Mahidol University, Bangkok 10700, Thailand; thanawat.phd@student.mahidol.ac.th (T.P.); sineewanlaya.wic@mahidol.ac.th (S.W.); maprangsompoppokakul@gmail.com (M.S.); virapong.pra@mahidol.ac.th (V.P.); chartchalerm.isa@mahidol.ac.th (C.I.-N.-A.); 2 Department of Clinical Chemistry, Faculty of Medical Technology, Mahidol University, Bangkok 10700, Thailand; napat.son@mahidol.ac.th

**Keywords:** uricase, coelacanth, *Latimeria*, disulphide bond engineering, hyperuricemia

## Abstract

The ideal therapeutic uricase (UOX) is expected to have the following properties; high expression level, high activity, high thermostability, high solubility and low immunogenicity. The latter property is believed to depend largely on sequence identity to the deduced human UOX (dH-UOX). Herein, we explored *L. menadoensis* uricase (LM-UOX) and found that it has 65% sequence identity to dH-UOX, 68% to the therapeutic chimeric porcine-baboon UOX (PBC) and 70% to the resurrected ancient mammal UOX. To study its biochemical properties, recombinant LM-UOX was produced in *E. coli* and purified to more than 95% homogeneity. The enzyme had specific activity up to 10.45 unit/mg, which was about 2-fold higher than that of the PBC. One-litre culture yielded purified protein up to 132 mg. Based on homology modelling, we successfully engineered I27C/N289C mutant, which was proven to contain inter-subunit disulphide bridges. The mutant had similar specific activity and production yield to that of wild type (WT) but its thermostability was dramatically improved. Up on storage at −20 °C and 4 °C, the mutant retained ~100% activity for at least 60 days. By keeping at 37 °C, the mutant retained ~100% activity for 15 days, which was 120-fold longer than that of the wild type. Thus, the I27C/N289C mutant has potential to be developed for treatment of hyperuricemia.

## 1. Introduction

Uricase or urate oxidase (EC 1.7.3.3; UOX) is a cofactor-free homotetrameric enzyme that catalyses the conversion of uric acid to 5-hydroxyisourate, an unstable intermediate that can undergo spontaneous or enzymatic hydrolysis to allantoin. This compound is far more soluble in water than uric acid, thus resulting in more efficient excretion from the body [1,2]. Most of the organisms in the three domains of life produce a functional UOX [3]. However, due to the accumulation of nonsense and missense mutations (pseudogenization) in the *uox* gene during evolution, a functional UOX is not present in hominoids, including human [4], putting these organisms at risk of hyperuricemia. In human, hyperuricemia is generally defined as the level of serum uric acid (SUA) greater than 6.8 mg/dL [5]. As a consequence, chronic hyperuricemia can lead to gout, kidney stone, kidney failure, hypertension and cardiovascular diseases [6]. Treatment of hyperuricemia and gout can be performed using xanthine oxidase inhibitors and uricosuric agents, which block uric acid synthesis and block reabsorption of uric acid at the kidneys, respectively [7]. Among these drugs, allopurinol, a member of xanthine oxidase inhibitors, is the most widely prescribed. However, usage of these drugs is not without problems; uricosuric agents are ineffective if renal function is impaired, allopurinol can induce allergic reactions and severe hypersensitivity [8] and refractory gout was reported to associate with patients receiving allopurinol [9]. In addition, these agents reduce SUA level very slowly, therefore, the drugs are inappropriate for treatment of acute hyperuricemia due to tumour lysis syndrome (TLS), which usually presents with very high level of SUA. Such high level of uric acid usually leads to precipitation of urate crystals in the renal tubules and eventually leads to acute kidney failure [10]. To address these problems, alternative treatment using UOX was investigated [11,12]. Based on its ability to rapidly clear SUA, UOX has become a promising agent for handling hyperuricemia [13,14]. Nowadays, two forms of clinically approved UOXs are available in the market; recombinant Aspergillus flavus UOX (rasburicase), which is approved for TLS [15] and PEGylated porcine-baboon chimera (PBC) UOX (pegloticase), which is approved for chronic refractory gout [16]. However, clinical application of these UOXs still faces with several problems. Rasburicase has a short serum half-life (16–20 h) [17] and its fungal origin contributes to its potent immunogenicity [18]. Similarly, pegloticase is also associated with anaphylaxis and infusion reactions [16] and production of antibody against PEG moiety was observed [19]. Nevertheless, as compared to rasburicase, immunogenicity of pegloticase is significantly reduced. This is believed to be due, in part, to high sequence similarity of pegloticase to the deduced human UOX (dH-UOX). This hypothesis is supported by the experiments with chimeric canine-human UOX [20] and porcine-human UOX, which showed reduced immunogenicity [21]. Therefore, this evidence drives the medical community to search for a more “human-like” UOX for treatment of hyperuricemia. Recently, genes involving purine catabolic pathway, including *uox*, of Indonesian coelacanths, *Latimeria menadoensis*, was characterized [22]. Based on analysis using protein BLAST search [23], we found that *L. menadoensis* UOX (LM-UOX) has 65% amino acid sequence identity to the dH-UOX [4], 68% to the therapeutic PBC UOX and 70% to mammalian (euarchontoglires) ancestor UOX [24]. Thus, the LM-UOX has potential to be developed for treatment of hyperuricemia. 

Herein, cDNA encoding the LM-UOX was cloned and expressed in *E. coli*. The enzyme was purified to more than 95% homogeneity and biochemical properties were characterized. In addition, it has been known that UOXs from higher organisms have less enzymatic activity and less structure stability [24,25,26,27]. Thus, to improve stability of the LM-UOX, replacement of targeted residues with cysteine to promote inter-subunit disulphide bridges, by site-directed mutagenesis, was performed. Among the generated mutants, I27C/N289C mutant with 120-fold increase in half-life at the body temperature (37 °C) was obtained. 

## 2. Results

### 2.1. Multiple Sequence Alignment (MSA) and Phylogenetic Analysis

**Figure 1:**
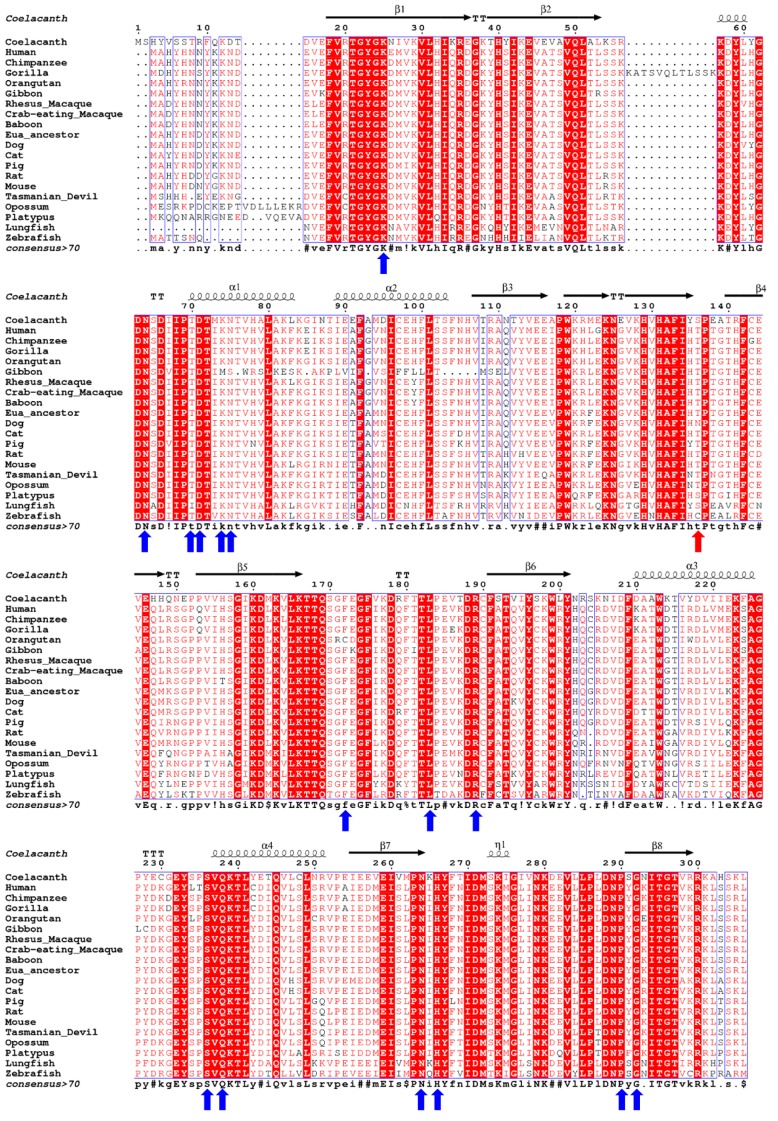
Multiple sequence alignment of coelacanth uricase (UOX) with UOX from related organisms. The secondary structure shown on top of the sequences is predicted by ESpript 3.0 [29] using PDB ID: 4MB8 as a template. Helices and strands are named according to Retailleau et al [28]. Consensus amino acids are shown under the sequences. Residues related to the active sites are indicated with blue arrows. Zebrafish C129, which forms natural disulphide bridges is indicated with a red arrow. Cat UOX sequence is started at 27th amino acid. The human sequence is deduced by replacing the two stop points (caused by the two premature stop codons 33 and 187) with arginine.

Translation of *L. menadoensis* mRNA for uricase (GenBank: HF678434.1) resulted in a protein sequence with 306 amino acids. Analysis of this protein sequence (LM-UOX) using blastp [23] showed that the sequence has 100% and 99% identity to *L. chalumnae* uricase isoform 1 (XP_005993907.1) and isoform 2 (XP_014342635.1), respectively. Interestingly, the LM-UOX had 65%, 68% and 70% amino acid sequence identity to the dH-UOX, the therapeutic PBC UOX and the mammalian (euarchontoglires) ancestor UOX, respectively. The last UOX, also known as An19/22, is a resurrected enzyme and the only UOX form higher organism with known 3D structure (PDB ID: 4MB8). MSA of uricase from 20 selected organisms including human, gorilla, chimpanzee, orangutan, gibbon, rhesus macaque, crab-eating macaque, hamadryas baboon, euarchontoglires ancestor, dog, cat, pig, mouse, rat, opossum, Tasmanian devil, platypus, coelacanth, lungfish and zebrafish revealed several conserved regions throughout the protein (white letters on red background, Figure 1). Fifteen residues localized close to the active site in *Aspergillus flavus* uricase [28] are highly conserved in the aligned sequences (indicated with blue arrows in Figure 1), except for the substitutions of T70I in African lungfish, K74M and N75S in gibbon and F172C in orangutan (coelacanth numbering).

**Figure 2:**
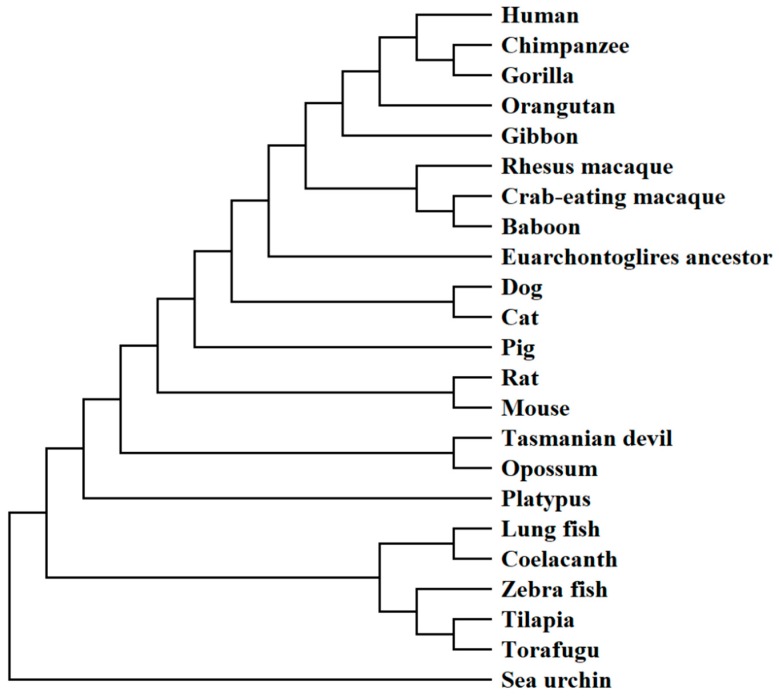
Phylogenetic tree of vertebrate UOX rooted with the ortholog of sea urchin. The tree is generated with Molecular Evolutionary Genetics Analysis (MEGA) X by using the neighbour-joining method.

Active site residues, T70, F172, R189, N264 and Q238 are highly conserved among functional UOX. Noteworthy, C-terminus of LM-UOX contains “SLK” residues, which is a putative peroxisomal translocation signal 1 (PTS1), indicating that the enzyme may localized in peroxisome. The phylogenetic tree was constructed by based on the uricase sequences of 23 selected organisms including primates, ferungulates, rodents, marsupials, platypus, lobe-finned fish, ray-finned fish and sea urchin. The UOX sequence of the last organism was considered as an outgroup and used to root the tree (Figure 2). The analysis split the vertebrate into the two separate groups of mammals and fish. In fish lineage, coelacanth formed a branch with lungfish representing a group of lobe-finned fish, which was clearly separated from a group of ray-finned fish. In mammal lineage, the primates were split into two separate groups of hominoids and cercopithecines, which represents a group with non-functional and functional uricase, respectively.

### 2.2. Homology Modeling and Quality Evaluation of the Predicted Models

**Figure 3:**
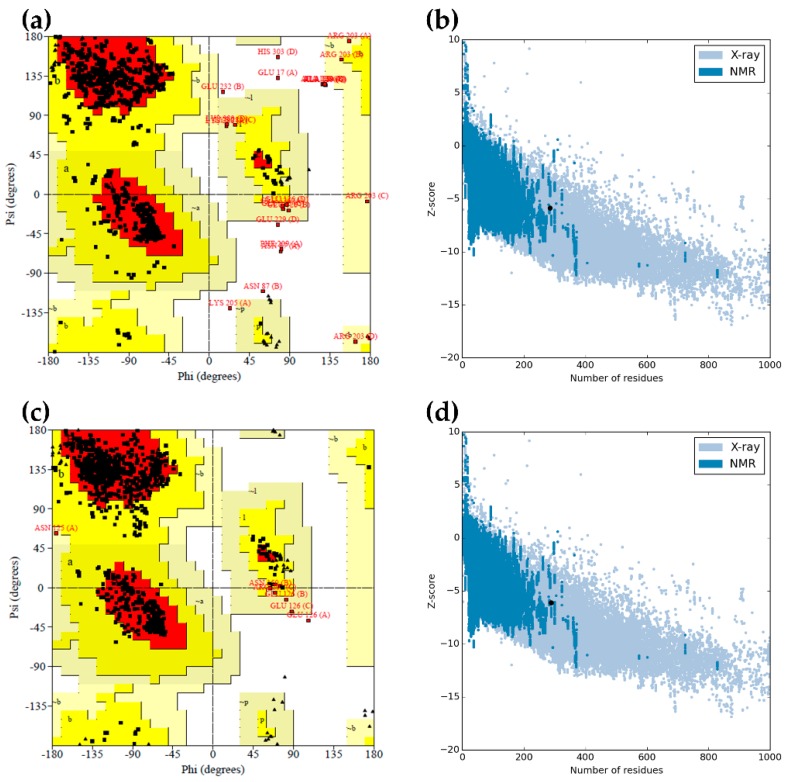
Evaluation of the predicted LM-UOX 3D models. Ramachandran plots of model 1 (**a**) and model 2 (**c**) and ProSA Z-score plots of model 1 (**b**) and model 2 (**d**) are shown. Ramachandran plot shows residues in the favoured regions (red), allowed regions (yellow), generously allowed regions (light yellow) and disallowed regions (white). ProSA Z-score plot shows Z-score (black dot) in a plot that contains the Z-scores of all experimentally determined protein chains currently available in the Protein Data Bank. Blue colour region represents Z-scores of protein structures characterized by NMR analysis and grey colour region represents Z-scores of protein structures characterized by X-Ray diffraction studies.

Based on the SWISS-MODEL template library search, more than 70 templates were found. However, only the top two templates, PDB ID: 4MB8 and 5M98, with >65% sequence identity to LM-UOX were selected. Thus, two models, which based on 4MB8 and 5M98, were generated. The Qualitative Model Energy ANalysis (QMEAN) Z-score of the predicted models was analysed and shown in Table 1. The value of > −3 indicates good quality of the predicted model. The structural stability and correctness of the predicted protein models were further evaluated using different tools (Table 1). Secondary structure of the predicted protein models was evaluated by Ramachandran Plot generated through PROCHECK. Model 1 (based on 4MB8) and model 2 (based on 5M98) showed 88.4% and 90.7% of the amino acid residues in favoured regions and 1.2% and 0.1% of the residues in disallowed regions, respectively (Figure 3a,c). Pro-Q was used to evaluate the models by checking the residue wise local quality of a model structure. The resulting LG-score of > 2.5 and the MaxSub-score of > 0.5 form Pro-Q indicate very good quality of the predicted models. Evaluation of the protein tertiary structure by matching with the statistics of the available experimentally determined structures using ProSA was also performed. The results showed that the ProSA Z-scores of the two models are within the range of experimentally determined native like protein structures (Figure 3b,d). Evaluation of the quality of tertiary structure by checking the residue wise compatibility of amino acid to the whole protein was performed by Verify3D. This program measures 3D–1D profile score for each amino acid residue. The protein model possessing more than 80% of amino acids with 3D–1D profile score ≥ 0.2 is consider to be stable. Analysis using Verify3D revealed that 78.31% and 81.23% of amino acids of model 1 and model 2 had 3D–1D profile score ≥ 0.2, respectively. Taken together, these results from various validation methods indicated that model 2 is more reliable than model 1. Therefore, only model 2 was selected for further analysis and studies.

**Figure 4:**
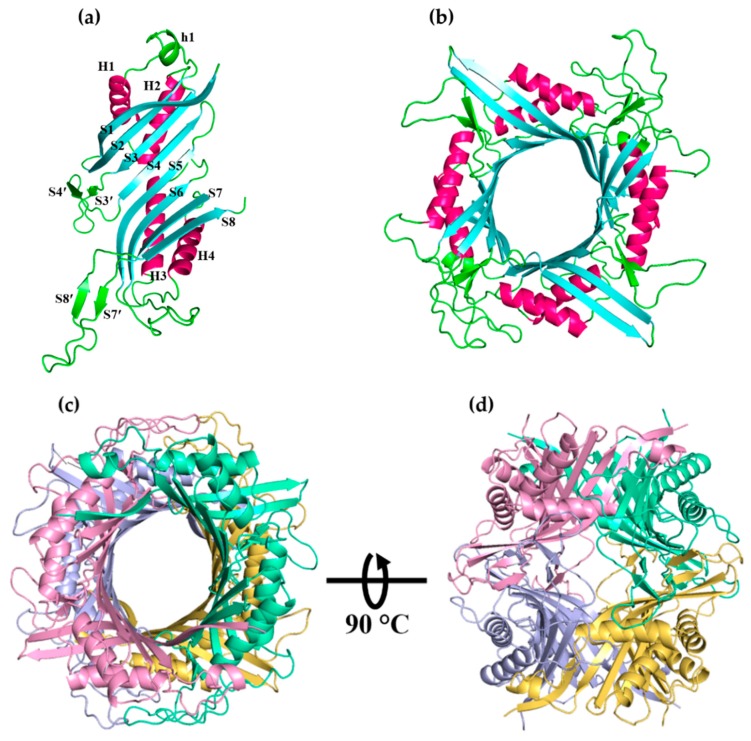
Predicted structure of LM-UOX. (**a**) The monomer with 4 helices in pink and 8 sheets in cyan. (**b**) The dimer. (**c**) Top view of the tetramer. (**d**) Side view of tetramer.

As visualized by PyMol, the predicted LM-UOX (model 2) has the typical homotetrameric uricase structure. One subunit is composed of two T-fold (tunnelling-fold) domains. Each domain has 4 anti-parallel beta-sheets and two alpha-helices on the concave side of the sheets. As shown in Figure 4a, connection of the two domains resulted in a subunit with 8 sequential anti-parallel beta-sheets (S1-S8) and four alpha-helices on the concave side of the sheets (H1–H4). Additionally, there are four short beta-sheets (S3′, S4′, S7′, S8′) and one short alpha-helix (h1). Association of the beta-sheet S1 to the beta-sheet S8 of the other subunit results in a dimeric structure (Figure 4b). Stacking of two dimers face-to-face results in a functional tetrameric structure (Figure 4c–d). The active sites are located at the dimer-dimer interface composing of T70 from one subunit and F172, R189, N264 and Q238 from the other subunit.

### 2.3. Prediction of Potential Disulfide Bridges 

**Figure 5:**
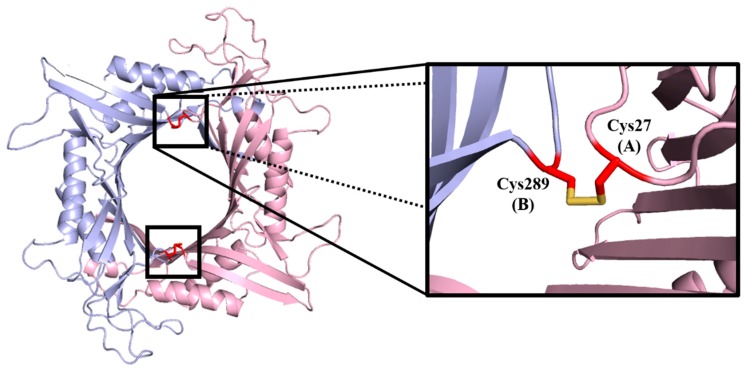
Inter subunit disulphide bridges of I27C/N289C mutant predicted by Disulfide by Design 2.0^TM^. In one dimer, one disulphide bridge links C27 of subunit A to C289 of subunit B while the other disulphide bridge links C27 of subunit B to C289 of subunit A.

Prediction of potential disulphide bridges was performed by the program Disulfide by Design 2.0. By employing the predicted LM-UOX structure as a template, 34 potential residue pairs were suggested. After evaluation of the parameters such as energy and χ3 value, only four pairs of residues were finalized and proceed for site-directed mutagenesis (SDM). These residue pairs include D288, N289, I27/N289 and A225/A132. Most of the selected residues are highly conserved (except for S136 and I27) but not related to the active site (except for N289). As shown in Table 2, some residue pairs promote disulphide bridges between subunits of the same dimer (A-B and C-D chains) while some residue pairs promote disulphide bridges between subunits of different dimers (A-C and B-D or A-D and B-C chains). In addition, as a feature of the program Disulfide by Design 2.0, the protein structure with the predicted disulphide bridges can also be viewed. An example of the predicted model is shown in Figure 5. Beside the selected residue pairs, since S136 is identical to C129 of zebrafish UOX, which forms natural inter-subunit disulphide bridges, SDM of S136C was also performed.

### 2.4. Protein Expression and Purification

**Figure 6:**
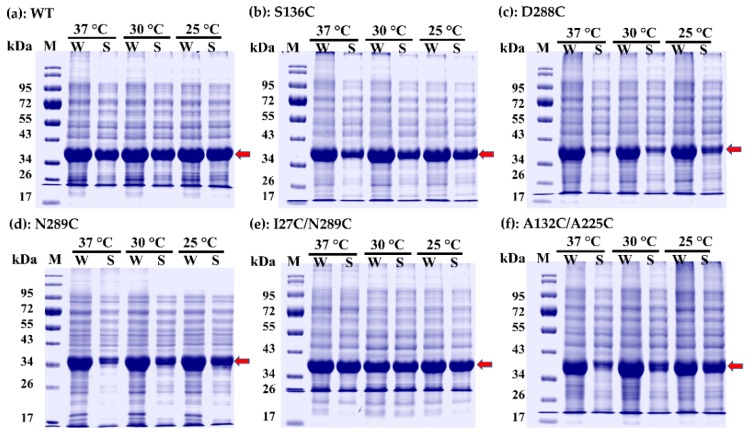
Expression of wild type LM-UOX and mutants in *E. coli*. Expression was done at 25 °C, 30 °C and 37 °C then whole cell lysate (W), which contains both soluble and insoluble protein fractions and soluble extract (S), which represents only soluble protein, were prepared and run on SDS-PAGE. **a**–**f** represent wild type, S136C, D288C, N289C, I27C/N289C and A132C/A225C, respectively. Red arrows indicate the overexpressed target proteins.

**Figure 7:**
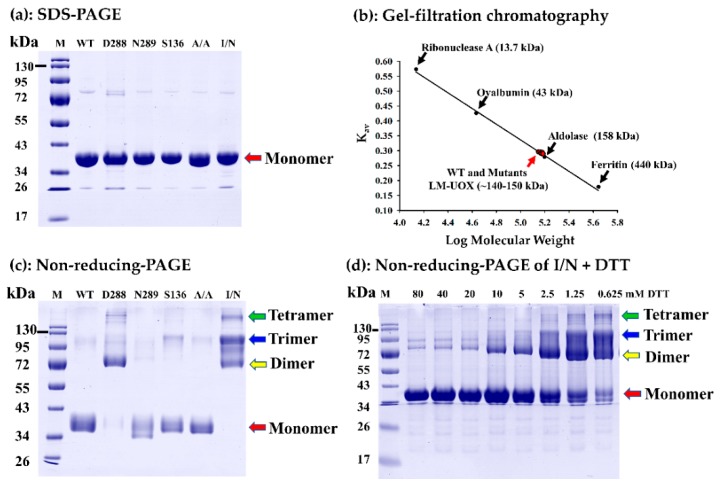
Investigation of protein molecular weight under denaturing and non-denaturing conditions. (**a**) SDS-PAGE shows purity after purification and molecular weight of all proteins under denaturing condition. (**b**) Gel-filtration chromatography calibration curve and the K_av_ values of WT and mutants (red circles) obtained from the experiments showing that all proteins have molecular weight ~140–150 kDa (**c**) non-reducing-PAGE shows the characteristics of proteins in the absence of β-mercaptoethanol; D288C and I27C/N289C did not dissociated into monomer, indicating the presence of special linkage between subunits, which could be disulphide bonds. (**d**) Dose dependent effect of disulphide bond reducing agent, DTT, on dissociation of I27C/N289C into monomer, confirming the presence of disulphide bonds.

To find the best protein expression condition, transformants were grown at different temperatures and agitations, which are 25 °C with shaking at 120 rpm, 30 °C with shaking at 150 rpm and 37 °C with shaking at 180 rpm. Whole cell lysate and crude extract, which were regarded as total and soluble protein, respectively, from each expression condition were run parallelly in SDS-PAGE. Expression of WT, S136C, D288C, N289C, I27C/N289C and A132C/A225C mutants are shown in Figure 6a–f, respectively. As explained in “Materials and methods”, the target protein is expressed with hexa-histidine tag (6His) at C-terminus. The tag is linked to the protein by lysine and leucine residues, which are originated from *Xho*I restriction site. As calculated by ExPASy Compute pI/Mw tool (https://web.expasy.org/compute_pi/), the LM-UOX-6His and all mutants had approximately the same molecular weights at 36.4 kDa (monomer). These molecular weight values were well correlated with the results presented in Figure 6 and Figure 7a, which shown the over-expressed/purified protein band at between 34 and 43 kDa (red arrows). 

Expression levels of each protein (WT and all mutants) at different culture temperatures were not significantly different. Similarly, solubility of WT, S136C and I27C/N289C mutants were unaffected by different culture temperatures. However, solubility of D288C, N289C and A132C/A225C mutants were obviously affected. These mutants were best soluble at 25 °C while less soluble at higher temperatures. Based on the amount of soluble recombinant protein expressed at 25 °C, maximum expression level was found in WT following by I27C/N289C, A132C/A225C, S136C, N289C and D288C, respectively. According to these results, expression of WT and all mutants for further purification was done at 25 °C with shaking at 120 rpm. WT and all LM-UOX mutants were purified to more than 95% homogeneity using Ni-NTA IMAC followed by gel-filtration chromatography (Figure 7a). In addition, based on retention volume from gel-filtration chromatography, molecular weight under native condition of each protein was also calculated. The results showed that all proteins possess natural molecular weights at ~145–150 kDa (Figure 7b), indicating that all proteins naturally exist in tetrameric form. The yield of protein purified from one-litre culture of WT and each mutant are shown in Table 3. Beside these experiments, since inter-subunit disulphide bonds were predicted in the I27C/N289C mutant, stabilization of the enzyme subunits was also studied. Exposure of these purified proteins to β-mercaptoethanol through SDS-PAGE experiment resulted in dissociation of tetramer to monomer (Figure 7a). Interestingly, through non-reducing PAGE (SDS-PAGE with no β-mercaptoethanol), the I27C/N289C mutant was found to retain in tetrameric, trimeric and dimeric forms but not monomeric form (Figure 7c). Beside I27C/N289C, D288C was also found to mostly exist in dimeric form and slightly exist in other forms. These results indicated the presence of inter-subunit disulphide bridges in I27C/N289C and D288C molecules. To prove this, I27C/N289C mutant was incubated with various concentrations of DTT (0.625–80 mM) for 30 min then the proteins form all conditions were run on non-reducing PAGE. The result showed that higher concentrations of DTT cause more dissociation of the protein into monomer (Figure 7d). Altogether, these results strongly indicated the existence of disulphide bridges in the I27C/N289C mutant.

### 2.5. Enzymatic Activity and Kinetic Parameters

**Figure 8:**
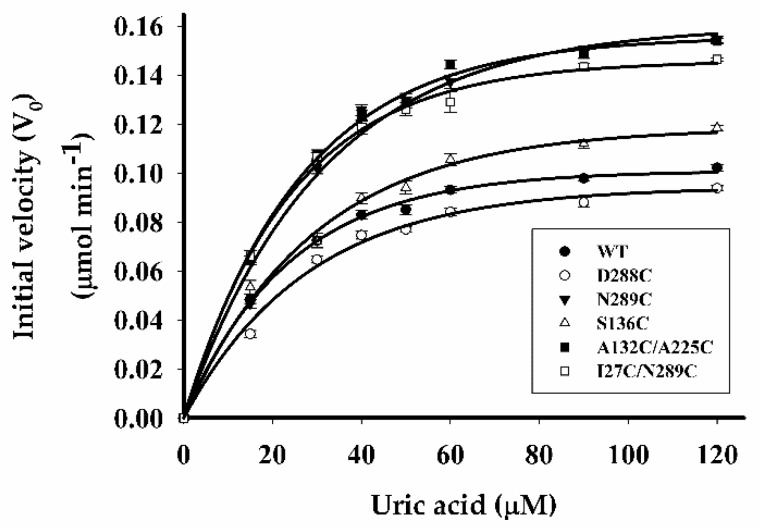
Comparison of enzymatic activity. The rate of substrate oxidation was calculated by based on a linear decrease in absorbance of uric acid. The plots show dependence of the initial rate of oxidation on uric acid concentration of wild type and mutants, which were fitted to the Michaelis-Menten equation.

Purified WT LM-UOX and all mutants were evaluated for catalytic rates at increasing substrate concentrations. Specific activity versus substrate concentration curves with Michaelis Menten fits are shown in Figure 8. WT LM-UOX possessed specific activity up to 10.45 unit/mg protein. Most mutants exhibited nearly the same specific activity except for A132C/A225C and S136C, which had ~0.5-fold increase and ~0.25-fold decrease in specific activity, respectively. The kinetic parameters (*K_m_* and *k_cat_*) of WT and mutants were calculated by least-squares analysis from Lineweaver-Burk plots of substrate’s concentrations against reaction velocity (Table 3). As compared with WT, most mutants exhibited 2-fold increase in apparent *K_m_* except for S136C and D288C, which had nearly the same and ~10-fold increase in *K_m_*, respectively.

Turnover number (*k_cat_*) of most mutants were found to be approximately the same as that of WT except for D288C and A132C/A225C, which exhibited ~2-fold increase in *k_cat_*. Calculation of the catalytic efficiency (*k_cat_*/*K_m_*) revealed that S136C and A132C/A225C have approximately the same efficiency as that of WT while D288C, N289C and I27C/N289C have 0.8-fold, 0.3-fold and 0.45-fold decrease in kinetic efficiency, respectively.

### 2.6. Characterization of WT LM-UOX and Mutants

**Figure 9:**
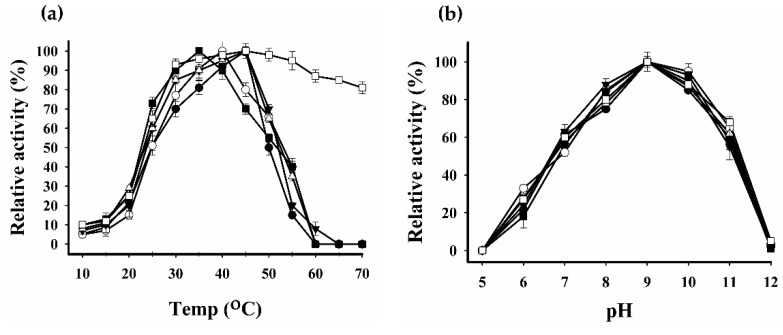
Effect of pH and temperature on uricase activity. Purified enzymes were assayed under different pHs (**a**) and temperatures (**b**) as described in “Materials and methods” section.

The effects of pH and temperature on enzymatic activity are shown in Figure 9a,b, respectively. The WT LM-UOX exhibited a maximum activity at pH 9 and 45 °C. Similarly, all mutants showed maximum activity at the same pH as that of WT. However, optimum temperatures of mutants were different. D288C, A132C/A225C and the rest of mutants exhibited optimum temperatures at 35 °C, 40 °C and 45 °C, respectively. The activity of WT and most mutants was rapidly lost at temperature higher than 45 °C. Interestingly, I27C/N289C retained activity up to 81% at 70 °C while the activity of WT and other mutants was completely abolished at this temperature.

### 2.7. Stability of WT LM-UOX and Mutants

**Figure 10:**
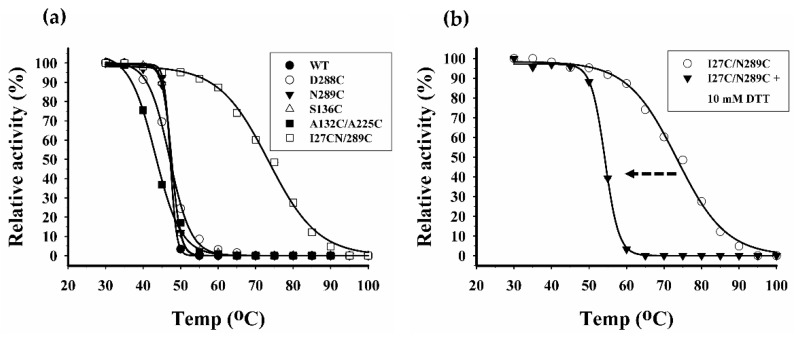
Plots of thermal inactivation after 2 min incubations at various temperatures. (**a**) Thermal inactivation of wild type and all mutants. (**b**) Thermal inactivation of I27C/N289C mutant in the absence and presence of 10 mM DTT.

**Figure 11:**
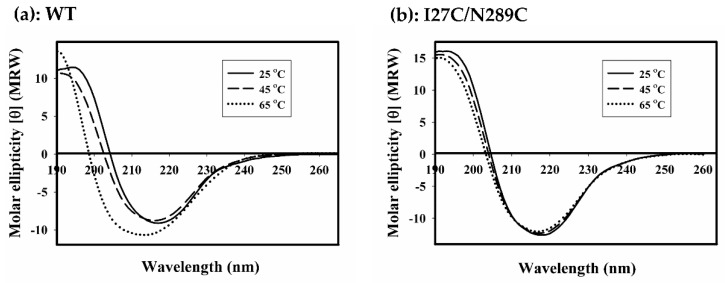
Circular dichroism spectra of wild type LM-UOX (**a**) and I27C/N289C mutant (**b**) at 25 °C, 45 °C and 65 °C.

To investigate the effects of temperatures on enzyme stability, WT and all mutants were heated for 2 min at different temperatures then residual activity was assayed. The residual activity plots were fitted with Boltzmann equation as shown in Figure 10a. Based on this equation, the temperature at which the enzyme is half-denatured (*T*_1/2_) can be inferred. Most mutants including WT retained almost 100% activity after heating at 45 °C; however, at higher temperatures, the activity was rapidly lost. N289C and A132C/A225C began to lost activity since 40 °C and the activity was completely lost at 60 °C. The *T*_1/2_ of WT, S136C, D288C, N289C and A132C/A225C were determined to be 47 °C, 47 °C, 47 °C, 47 °C and 43 °C, respectively. Interestingly, I27C/N289C mutant retained more than 80% activity after heating at 60 °C. At increasing temperatures, the activity was gradually lost and completely lost at 90 °C. The *T*_1/2_ of I27C/N289C mutant was found to be 75 °C, which is 28 °C higher than that of WT. This result indicated that I27C/N289C is the only mutant with improved thermostability. To further investigate the temperature dependence of the secondary structure of WT and I27C/N289C mutant, CD spectra of the proteins incubated at 25 °C, 45 °C and 65 °C were recorded. As shown in Figure 11a, spectra of WT collected at 25 °C and 45 °C were very similar. At these two temperatures, the negative ellipticity peaks were at 217 nm and 216 nm and the positive ellipticity peaks were at 194 and 191 nm, respectively.

The peak at ~216 nm is commonly associated with beta-sheet secondary structure. However, the spectra measured at 65 °C showed distinct molar ellipticity between 190–220 nm, indicating secondary structure alteration. The spectra showed a negative ellipticity peak at 214 nm and a positive ellipticity peak at 190 nm. Conversely, CD spectra of I27C/N289C mutant collected at 25 °C, 45 °C and 65 °C were hardly different (Figure 11b); the negative and positive ellipticity peaks of the three spectra were at 217 nm and 191 nm, respectively.

**Figure 12:**
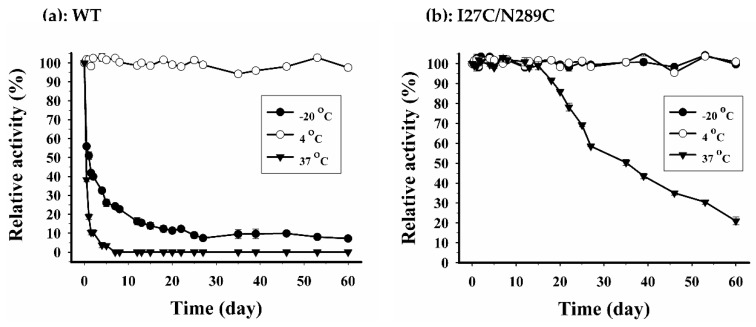
Effects of storage temperatures on residual activities of wild type LM-UOX (**a**) and I27C/N289C mutant (**b**).

This result indicated that the secondary structure of I27C/N289C mutant is hardly affected at 65 °C. Noteworthy, secondary structure alterations at various temperatures seen by CD analysis were well correlated with the above findings of heat inactivation (Figure 10a). Altogether, these results imply that disulphide engineering may be successful in I27C/N289C mutant. To prove this hypothesis, I27C/N289C mutant was incubated with 20 mM DTT (1,4-dithiothreitol) for 30 min to reduce disulphide bond then the protein was subjected to heat inactivation as described above. As shown in Figure 10b, after treatment with DTT, *T*_1/2_ of I27C/N289C mutant decreased from 75 °C to 54 °C. This effect of DTT further supported the existent of disulphide bonds in I27C/N289C mutant. In addition, we have also investigated the stability of WT and I27C/N289C mutant under storage temperatures (−20 °C, 4 °C and 37 °C). The residual activity was assessed over 60 days period and the results are shown in Figure 12a,b. At 4 °C, both WT and I27C/N289C mutant retained almost 100% of the activity for over 60 days. At −20 °C, the relative activity of WT rapidly dropped to 50% since day 1 while I27C/N289C mutant retained nearly 100% of activity throughout the measuring period. Interestingly, at 37 °C, the I27C/N289C mutant retained nearly 100% of activity 15 days while the WT lost more than 80% of activity since day 1. This result also supports the improved stability of the I27C/N289C mutant. 

## 3. Discussion

Study on treatment of hyperuricemia in human using uricase (purified from beef kidneys [30]) was first reported in 1957 [13]. In 1967-1968, uricase from *Aspergillus flavus* (AF-UOX) was extracted [31] and used for treatment of hyperuricemia in animal and human [32]. Due to the ability to rapidly reduce serum uric acid, UOX has become a promising agent for handling hyperuricemia. In 1975, the AF-UOX was first approved in France for treatment of hyperuricemia caused by TLS. However, owing to its fungal origin, administration of the drug was frequently associated with allergy and hypersensitivity. To avoid these side effects, a recombinant version of AF-UOX produced in *S. cereviaceae* was developed [33]. Nevertheless, this new version of AF-UOX was still a fungal enzyme; its immunogenicity was not that much reduced [18]. In addition to development of microbial enzymes, vertebrate enzymes were also studied. PEGylated porcine-baboon chimera (PBC) UOX has been produced and subsequently approved for treatment of chronic refractory gout [16]. Beside PEGylation, the baboon sequence, which has high sequence identity to the dH-UOX was believed to help avoiding immune system while the porcine sequence was believed to contribute high enzymatic activity [21]. Based on this summation, UOXs with high sequence identity to the dH-UOX were believed to contribute less immunogenicity. However, UOXs from higher primates such as hominoids are inactive and UOXs from other higher mammals are usually presented in a crystalloid form in lysosome [25,26,27], indicating low structural instability, which results in a tendency to precipitate and reduced enzymatic activity. This behaviour prevents recombinant production of these enzymes in soluble and active form. Moreover, it has been widely accepted that one of the highest risk factors for drug immunogenicity is the property of proteins to aggregate [34]. Thus, to apply the aggregated UOX for therapeutic application, the enzymes must go through several complicated refolding steps in manufacturing process and there is no guarantee for the protein to re-aggregate. This situation can also be seen in production of an unnatural PBC UOX [35]. Therefore, several attempts have been made to develop an ideal therapeutic uricase with improved properties, for example, soluble expression, neutral pH solubility, high level of expression in conventional host such as *E. coli*, high thermal stability and high activity.

Coelacanth (*Latimeria* spp.) is an ancient lobe-finned fish previously known from fossils and believed to have been extinct since 70 million years ago [36]. The morphology of the living fish is similar to that of fossils that date back ~300 million years. These findings lead to the hypothesis that evolution of this fish must be very slow [36,37] and the fish could be one of the nearest extant relatives to our last fish ancestor, the first fish that crawled up on land [38]. Study of their genes or proteins may provide insights into evolution of our ancestors. Beside studying living fossil like coelacanth, specific genes or proteins of ancient organisms may also be resurrected by evolutionary models. Recently, UOX of ancient mammals (euarchontoglires ancestor), which lived before hominoids *uox* gene pseudogenization event, were resurrected and used to study the evolutionary history of primate uricase [24]. In addition, one of the resurrected enzymes, An19/22, was tested for pharmacokinetics in rat model. The result showed that serum UOX activity of An19/22 was 100-fold more stable than non-PEGylated PCB. Due to its high catalytic activity, high sequence identity to the deduced human UOX and long serum half-life, the authors suggested that An19/22 UOX has potential therapeutic value as a non-PEGylated protein, which is important because human patients develop antibodies to the PEG moiety on pegloticase [39].

In the present study, based on UOX protein sequence, we have analysed the evolutionary relationships of 23 selected organisms (Figure 2) and found that coelacanth UOX (LM-UOX) is more closely related to mammal UOX than other fish UOX, except for lungfish. However, as shown in Figure 1, amino acids prior to N15 of lungfish UOX are missing, conclusions drawn from experiments using this sequence may not be reliable. Interestingly, based on sequence alignment shown in Figure 1, LM-UOX has ~70% sequence identity to An19/22, indicating that LM-UOX may have similar properties especially pharmacokinetics to that of An19/22, which has potential to be developed for treatment of hyperuricemia. To study biochemical properties of LM-UOX, we cloned and expressed LM-UOX in *E. coli*. As shown in Figure 6a, the WT LM-UOX was overexpressed in soluble form at all tested temperatures, which is promising for further downstream processes. Purification of the recombinant protein from soluble crude extract showed that 1-L culture of *E. coli* yielded the protein up to 132 mg (Table 3), which is a reasonably high amount of recombinant protein. However, this yield can be maximized in industrial scale using well-equipped fermenters or bioreactors. Investigation of enzymatic activity and kinetic parameters showed that the protein has specific activity 4-fold greater and *K_m_* 4-fold smaller than that of An19/22. Taken together, these results indicated that LM-UOX has high potential for further development as therapeutic UOX. However, as previously mentioned, UOX form higher organism usually suffers from structure instability. We then evaluated thermostability and effects of storage temperature on WT LM-UOX, which we found that the protein can tolerate heating at 45 °C for only 2 min and can tolerate 37 °C temperature for only 7 h. Thus, the protein may not stable enough for drug formulation, storage and administration. To improve its thermostability, PEGylation [40] and protein engineering [41] may be applied. However, production of antibody against PEG moiety was reported; thus, in this study, protein engineering was selected. Prior to engineering, we have modelled 3D structure of WT LM-UOX using Swiss-Model and applied the best model for further designing of disulphide bridges using Disulfide by Design 2.0^TM^. In modelling process, based on template search result, the best template with maximum scores was 4MB8 followed by 5M98, which are the structure of An19/22 and zebrafish UOX, respectively. However, after modelling, best validation scores were found in modelled structure that used 5M98 as a template (Table 1). This may be due, in part, to the quality of the template as seen by Ramachandran plot of the two templates. The program Disulfide by Design 2.0^TM^ suggested more than 30 residue pairs with potential inter-subunit disulphide bridges. However, only 4 residue pairs with good prediction scores and not related to critical part of enzyme, such as active site, were selected. Beside these residue pairs, as mentioned above, S136 was also selected. All mutants were created by SDM, expressed in *E. coli*, purified to more than 95% homogeneity (Figure 7a) and characterized for enzymatic activity (Figure 8) and thermostability (Figure 10a). Mutation of D288, N289 and A132/A225 to cysteine severely affected protein stability. This conclusion was drawn from notification these proteins are associated with poor solubility (Figure 6c,d), low purification yield (Table 3) and reduced thermostability. These results suggested that these residues may play an important role in structure stabilization. Interestingly, when N289C was co-introduced with I27C, the enzyme structure is stabilized, indicating the formation of disulphide bridges. Notably, S136C did not change protein stability nor enzymatic activity. Together with observation that S136 is not a conserved residue, this result confirmed that S136 play neither role in enzyme stability nor activity. In addition, this result reminds that the attempt to introduce disulphide bridges by mutation of identical residues, presented in the ortholog proteins, to cysteine does not always success.

One desirable property of therapeutic proteins is the ability to tolerate temperatures during formulation, manufacture, storage, transport, handling and patient administration [42]. In this study, we evaluated stability of the proteins at −20 °C, 4 °C and 37 °C for 60 days. Although WT LM-UOX retained almost 100% activity for 60 days at 4 °C, its half-life at −20 °C and 37 °C were less than 1 day, indicating that the protein may not survive storage and patient body temperatures. Conversely, I27C/N289C mutant retained almost 100% activity for at least 15 days at all tested temperatures and retained almost 100% activity for at least 60 days at −20 °C and 4 °C. These results indicated that, without further modifications, I27C/N289C mutant may perfectly survive all temperatures from manufacturing till patient administration.

## 4. Materials and Methods

### 4.1. Bacterial Strains, Plasmid and Chemicals

All bacterial strains and pET20b(+) plasmid were obtained from Novagen (EMD Bioscience, Madison, WI, USA). *E. coli* strain Novablue and BL21(DE3) were used as cloning and expression hosts, respectively. *Nde*I and *Xho*I were from Fermentas (Thermo Fisher Scientific, Waltham, MA, USA). KOD-Plus DNA polymerase was purchased from Toyobo (Osaka, Japan). QuikChange Lightning site-directed mutagenesis kit was obtained from Agilent Technologies (Santa Clara, CA, USA). Uric acid and lithium carbonate were from Sigma-Aldrich (St. Louis, MO, USA). Concentration of uric acid was standardized using extinction coefficient of 12.6 × 10^−3^ M^−1^cm^−1^ at 293 nm.

### 4.2. Multiple Sequence Alignment (MSA) and Phylogenetic Analysis

Uricase protein sequence of rhesus macaque (XP_001103553.2), crab-eating macaque (XP_005542943.1), hamadryas baboon (AAA35395.1), euarchontoglires ancestor (4MB8_A), dog (NP_001011886.1), cat (XP_003990310.2), pig (NP_999435.1), mouse (NP_033500.1), rat (NP_446220.1), opossum (XP_007480672.1), Tasmanian devil (XP_023357490.1), platypus (XP_001509964.1), coelacanth (CCT61357.1), lungfish (AAZ92543.1), zebrafish (NP_001002332.1), tilapia (XP_005457392.1), torafugu (XP_003974322.1) and sea urchin (XP_003729237.1) were retrieved from NCBI GenPept. While the deduced uricase protein sequence of gorilla, chimpanzee, human, orangutan and gibbon were from the work previously published by Oda et al [4]. Protein multiple sequence alignment was performed using ClustalW [43]. The evolutionary history was inferred using the Neighbour-Joining method [44]. The optimal tree with the sum of branch length = 2.25634656 is shown. The tree is drawn to scale, with branch lengths in the same units as those of the evolutionary distances used to infer the phylogenetic tree. The evolutionary distances were computed using the Poisson correction method [45] and are in the units of the number of amino acid substitutions per site. This analysis involved 23 amino acid sequences. All ambiguous positions were removed for each sequence pair (pairwise deletion option). There was a total of 384 positions in the final dataset. Evolutionary analyses were conducted in MEGA X [46].

### 4.3. Homology Modelling 

The amino acid sequence of wild-type *L. menadoensis* uricase (LM-UOX) was obtained from UniProt (accession number of M3XGK0). The 3D structural model of LM-UOX was predicted by the Swiss-Model server [47] using the euarchontoglires ancestor (PDB ID: 4MB8) and the zebrafish (PDB ID: 5M98) uricase crystallographic structures as templates [24,48]. The resulting models were estimated for global and per-residue quality using the QMEAN scoring function [49] in the Swiss-Model server. The models were also evaluated for stereochemical quality by PROCHECK [50], Verify3D [51,52], ProSA [53] and Pro-Q [54]. The 3D structural visualization was performed on PyMOL [55].

### 4.4. Prediction of Potential Disulfide Bridges

Based on the predicted three-dimensional structure of LM-UOX, potential disulphide bridges were predicted using the web-based program Disulfide by Design 2.0 [56]. For software settings, only inter-chain prediction and the χ3 torsion angles at −87 or +97 ± 30 degrees were selected. Suggested residue pairs with energy less than 2.2 kcal/mol were further evaluated for possible involvement with substrate catalysis. Only residue pairs presented outside the catalytic centre were further proceeded for site-directed mutagenesis (SDM). In addition, in zebrafish uricase, a natural disulphide bond between subunit A and C is formed by C129. Since zebrafish C129 and coelacanth S136 are identical, coelacanth S136 was also selected for substitution with cysteine.

### 4.5. DNA Manipulations

*L. menadoensis* cDNA for uricase (LM-UOX) was synthesized by Integrated DNA Technologies (Skokie, Illinois). The gene was PCR-amplified using primers shown in Table 4 then PCR product was digested with *Nde*I and *Xho*I and cloned into pET20b(+) plasmid pre-treated with the same enzymes. The constructed plasmid was designated as pET20LM-UOX, which is used for expression of wildtype (WT) enzyme. SDM was performed according to manufacturer’s protocol, using primers listed in Table 4, generating plasmids encoding mutant enzymes including S136C, D288C, N289C, I27C/N289C and A132C/A225C. All constructed plasmids were subjected to automated DNA sequencing to verify the accuracy of cloning procedure and site-directed mutagenesis.

### 4.6. Protein Expression and Purification

Plasmid encoding WT and mutant LM-UOXs including S136C, D288C, N289C, I27C/N289C and A132C/A225C were individually transformed into *E. coli* BL21(DE3). To find the best condition for protein expression, each transformant was grown in 5 mL of terrific broth supplemented with 100 µg/mL ampicillin at 37 °C with shaking at 180 rpm. After A600 nm reached 1.0, cultures were supplemented with 1 mM Isopropyl β-D-1-thiogalactopyranoside (IPTG) to induce protein expression. Then cultures were continued for 16 h at 3 different conditions; (i) 25 °C with shaking at 120 rpm, (ii) 30 °C with shaking at 150 rpm and (iii) 37 °C with shaking at 180 rpm. Cells were harvested by centrifugation and resuspended in 50 mM Tris-HCl buffer pH 8.0 supplemented with NaCl 500 mM then cells were disrupted by ultrasonic disintegration. The resulting material was regarded as whole cell lysate. Half portion of the whole cell lysate were centrifuged at 27,216× *g* for 5 min then supernatant was collected. This portion was regarded as crude extract or soluble fraction. Then whole cell lysates and soluble fractions from each condition were analysed for overexpression and solubility by SDS-PAGE analysis. For protein purification, larger portion of crude extract was prepared from the best expression condition then the crude extract was filtered using 0.45 µm Minisart filter (Sartorius) to remove insoluble materials. Then, the crude extract was loaded onto Ni-nitrilotriacetic acid (Ni-NTA) agarose column (pre-equilibrated with 50 mM Tris-HCl buffer pH 8.0 supplemented with NaCl 500 mM) attached to ÄKTA pure protein purification system (GE healthcare life sciences, Sweden). The protein was eluted with gradient imidazole in the same buffer and further purified by 16/60 Sephacryl S-300 HR gel-filtration chromatography column (GE healthcare life sciences, Sweden). Fractions containing target protein were pooled and concentrated by 100K Amicon^®^Ultra-15 centrifugal filter devices (EMD Millipore Corporation, MA, USA). Pooled sample was centrifuged at 10,976× *g* for 5 min then the remaining sample in the device was purged using automatic pipette. Then these steps were repeated until the target volume is archived. Purified proteins were checked for purity and molecular weight under denaturing condition by SDS-PAGE analysis. Purified proteins were kept under −80 °C, in the presence of 20% glycerol until used. Protein concentration was determined by Bradford Assay (Bio-Rad).

### 4.7. Polyacrylamide Gel Electrophoresis

SDS-PAGE was performed according to the method described by Laemmli [57]. β-mercaptoethanol was omitted for non-reducing SDS-PAGE.

### 4.8. Uricase Activity Assay

Enzymatic activity measurements were carried out with a Shimadzu UV-2450 UV-visible spectrophotometer with a temperature-controlled cuvette-holder, at 30 °C. The activity was measured by following the decrease of absorbance of uric acid at 293 nm during the reaction. Uric acid was freshly prepared in 50 mM sodium borate buffer, pH 8.0. Kinetic constants were assayed by varying concentration of uric acid from 15 µM to 120 µM. The assay was performed in triplicate runs. Kinetic parameters were calculated by least-squares analysis from Lineweaver-Burk plots of substrate’s concentrations against reaction velocity. One unit of uricase activity is defined as the amount of enzyme required to convert 1 µmol of uric acid to allantoin per minute at 30 °C, pH 8.0.

### 4.9. Enzyme Characterization

Optimum pH was studied over a pH range of 4.0–12.0 using 50 mM sodium citrate buffer (pH 4.0–6.0) and 50 mM Tris-HCl buffer (pH 7.0–12.0). Optimum temperature was evaluated by measuring the uricase activities at temperatures ranging from 20–80 °C. The assay at each condition was performed in triplicate runs.

### 4.10. Thermostability Measurement

Thermostability was evaluated by incubating the enzyme over temperature range of 30–100 °C for 2 min then the residual activity was assayed. In addition, the enzyme was also stored at —20 °C, 4 °C and 37 °C for 60 days and residual activity was assayed during the storage period. The assay at each condition was performed in triplicate runs.

### 4.11. Circular Dichroism Spectroscopy

The circular dichroism (CD) spectra were recorded in a 1.0 mm quartz cuvette at 25 °C using a Jasco J-815 spectropolarimeter. The spectra are an average of five consecutive scans from 260 to 193 nm, recorded with a band width of 1 nm, a time constant of 8 s and a scan rate of 50 nm/min. Spectra were recorded and corrected by subtracting background contributions of blank samples. 

## 5. Conclusions

Recombinant wild type coelacanth uricase was successfully produced in soluble and active form. The purified enzyme had specific activity up to 10.45 unit/mg, which was about 2-fold higher than that of the therapeutic PBC UOX. Based on homology modelling, we further engineered a mutant, I27C/N289C, with inter-subunit disulphide bridges. The mutant had similar specific activity and kinetic parameters to that of wild type but its thermostability was dramatically improved. Up on storage at —20 °C and 4 °C, the mutant retained ~100% activity for at least 60 days. By keeping at 37 °C, the mutant retained ~100% activity for 15 days, which was 120-fold longer than that of wild type enzyme. Taken together with high sequence identity of LM-UOX to the dH-UOX, the therapeutic PBC UOX and the An19/22 UOX, the I27C/N289C mutant has potential to be developed for treatment of hyperuricemia.

## Figures and Tables

**Table 1:** Assessment of the predicted three-dimensional structures of LM-UOX.

Validation Index	Model 1 (Based on PDB ID: 4MB8)	Model 2 (Based on PDB ID: 5M98)
SWISS-MODEL		
GMQE	0.80	0.81
QMEAN	−1.55	−1.39
Ramachandran plot (PROCHECK)		
Residues in most favoured regions	88.4%	90.7%
Residues in additional allowed regions	9.4%	8.7%
Residues in generously allowed regions	1.1%	0.5%
Residues in disallowed regions	1.2%	0.1%
Pro-Q		
LG-score	6.915	7.339
MaxSub-score	0.647	0.711
ProSA Z-score	−5.85	−6.09
Verify3D (% of amino acids with average 3D-1D score ≥ 0.2)	78.31%	81.23%

**Table 2:** Characteristics of the predicted disulphide bonds.

Residue Pairs	Number of Disulphide Bridge/ Enzyme Molecule	Inter-Subunit Linkage
S136C	2	A-C and B-D
D288C	2	A-D and B-C
N289C	2	A-D and B-C
I27C/N289C	4	2(A-B) and 2(C-D)
A132C/A225C	4	2(A-C) and 2(B-D)

**Table 3:** Specific activity, kinetic parameters and production yield of WT and LM-UOX mutants.

LM-UOX and Variants	Specific Activity (unit/mg)	*K_m_* (µM)	*k_cat_* (s^−1^)	*k_cat_*/*K_m_* (µM^−1^ s^−1^)	Yield (mg/1-L Culture)
WT	10.45 ± 0.14	18.43 ± 0.4	29.89 ± 0.3	1.62	132.1
S136C	7.88 ± 0.08	15.28 ± 0.1	22.49 ± 0.1	1.47	130.0
D288C	9.38 ± 0.09	189.65 ± 0.2	65.07 ± 0.3	0.34	5.8
N289C	10.38 ± 0.10	30.46 ± 0.1	34.06 ± 0.1	1.12	13.2
I27C/N289C	9.75 ± 0.05	35.39 ± 0.1	32.58 ± 0.2	0.92	100.5
A132C/A225C	15.38 ± 0.12	39.19 ± 0.5	58.19 ± 0.6	1.49	40.5

Values are means ± standard deviation of the replicates.

**Table 4:** Primers for LM-UOX cDNA amplification and site-directed mutagenesis. *Nde*I and *Xho*I restriction sites were included in forward and reverse primers (underlined text), respectively.

Primers	Description	DNA sequence (5’→3′)
LM-UOX FP ^a^	for construction of pET20LM-UOX	GGTCTTCCATATGTCCCACTATGTTTCC
LM-UOX RP ^b^		CCGCTCGAGCAGTTTGGAATGTGCC
I27C FP	for mutagenesis of I27→C27	GGCTACGGGAAAAACTGCGTAAAAGTCTTGCAC
I27C RP		GTGCAAGACTTTTACGCAGTTTTTCCCGTAGCC
A132C FP	for mutagenesis of A132→C132	GCATGTGCATTGCTTTATTTACAGTCC
A132C RP		GGACTGTAAATAAAGCAATGCACATGC
S136C FP	for mutagenesis of S136→C136	GCATGCTTTTATTTACTGTCCAGAAGCAACTCGG
S136C RP		CGAGTTGCTTCTGGACAGTAAATAAAAGCATG
A225C FP	for mutagenesis of A225→C225	ATTGAGAAGTTTTGTGGCCCTTACGAGTG
A225C RP		CACTCGTAAGGGCCACAAAACTTCTCAAT
D288C FP	for mutagenesis of D288→C288	GTTCTGCTGCCTCTGTGCAACCCATCTGGCAAC
D288C RP		GTTGCCAGATGGGTTGCACAGAGGCAGCAGAAC
N289C FP	for mutagenesis of N289→C289	CTGCTGCCTCTGGACTGCCCATCTGGCAACATTAC
N289C RP		GTAATGTTGCCAGATGGGCAGTCCAGAGGCAGCAG

^a^ FP = Forward primer, ^b^ RP = Reverse primer.
